# Salmon bone-derived chondroitin sulfate nanogels for controlled delivery of curcumin: release behavior, structural characteristics and anti-inflammatory efficacy

**DOI:** 10.1039/d6ra06378k

**Published:** 2026-10-05

**Authors:** Quang Van Ngo, Nguyen Chi Nguyen, Hong Thi Pham, Cuong Duc Ho, Thu Thi Minh Quach, Thuy Thi Thu Thanh

**Affiliations:** a Institute of Chemistry, Vietnam Academy of Science and Technology 18 Hoang Quoc Viet, Nghia Do Hanoi 100000 Vietnam thuyttt@ich.vast.vn; b School of Chemistry and Life Science, Hanoi University of Science and Technology 01 Dai Co Viet, Bach Mai Hanoi 100000 Vietnam

## Abstract

Marine-derived polysaccharides have emerged as sustainable biomaterials for advanced drug delivery; however, the relationship between their molecular organization and drug release behavior remains insufficiently understood. In this study, chondroitin sulfate (CS) extracted from salmon bone was utilized to fabricate Ca^2+^-crosslinked nanogels for the controlled delivery of curcumin (Cur). The optimized nanogels exhibited a hydrodynamic diameter of 217.3 ± 15.6 nm, a polydispersity index of 0.25 ± 0.02, and a negative zeta potential of −26.9 ± 0.14 mV, indicating good colloidal stability. Comprehensive structural characterization *via* FT-IR and XRD indicated that the nanogel framework is stabilized by Ca^2+^-mediated ionic crosslinking and multi-point hydrogen bonding. Notably, XRD analysis revealed that while encapsulated curcumin primarily adopts an amorphous molecular dispersion, the emergence of a specific reflection at 2*θ* ≈ 14° suggests that a minor fraction adopts a locally ordered state stabilized within the nanogel microenvironment. The nanogels demonstrated a sustained biphasic release profile governed by anomalous (non-Fickian) diffusion (*n* = 0.711). Furthermore, in LPS-stimulated RAW 264.7 macrophages, the CS-Cur nanogels showed significantly enhanced anti-inflammatory efficacy and high biocompatibility compared to free curcumin. These findings highlight salmon bone-derived CS as a sustainable, high-performance matrix for hydrophobic drug delivery, providing new insights into the role of cargo-induced structural organization in regulating therapeutic release and biological performance.

## Abstract 2

Curcumin may contribute to nanogel structural organization, serving not only as a therapeutic payload.

## Graphical abstract

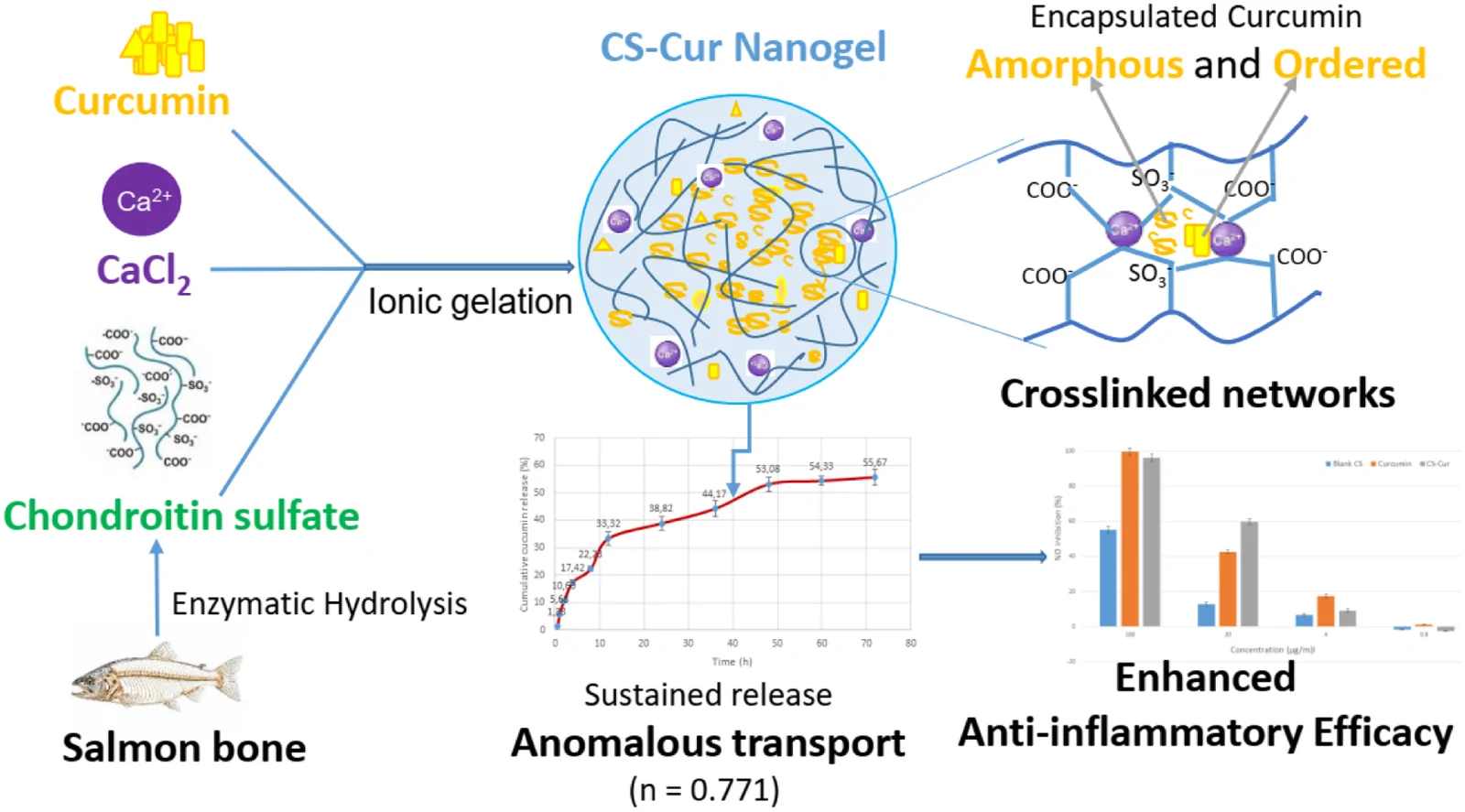

## 1 Introduction

Nanogels are three-dimensional hydrogel nanoparticles formed by physically or chemically cross-linked polymer networks. They uniquely combine the high water-holding capacity and structural flexibility of macroscopic hydrogels with the prolonged systemic circulation and enhanced cellular internalization typical of nanotechnology. Within this domain, biopolymers are widely investigated due to their excellent biocompatibility, low toxicity, and biodegradability, making them ideal scaffolds for therapeutic encapsulation.^1–4^ Concurrently, advanced supramolecular biomaterials and self-assembled polymeric or hydrogel networks have emerged as powerful platforms for drug delivery.^5–7^

Among biopolymers, chondroitin sulfate (CS), a naturally occurring sulfated glycosaminoglycan consisting of alternating β-d-glucuronic acid and *N*-acetyl-d-galactosamine units, is highly attractive for nanogel fabrication. The abundance of carboxylate and sulfate groups in CS facilitates robust ionic cross-linking, while its intrinsic biological activities offer synergistic therapeutic benefits in drug delivery systems.^8–13^

Despite these advantages, the challenge of stabilizing hydrophobic payloads like curcumin (Cur) within CS-based frameworks remains significant. While Cur possesses potent anti-inflammatory and antitumor properties, its poor aqueous solubility and rapid physiological degradation severely limit its clinical efficacy.^14,15^ Encapsulation within a nanogel framework is a well-established strategy to overcome these hurdles.^16^ However, the molecular dynamics of this process are often simplified. Specifically, the role of the therapeutic cargo as an active structural constituent, rather than a passive occupant, warrants deeper investigation.

While CS is widely sourced from terrestrial livestock (bovine and porcine), these traditional sources carry inherent biosafety concerns, including the potential transmission of zoonotic pathogens (such as bovine spongiform encephalopathy, BSE) and strict religious or dietary limitations.^17,18^ In contrast, utilizing seafood processing by-products, specifically salmon bone, offers a safe, sustainable, and economically circular bio-resource.^19,20^ More importantly, marine-derived CS may exhibit sulfation patterns and compositional heterogeneity distinct from those commonly reported for terrestrial sources.^21,22^

Consequently, this study aims to fabricate and structurally elucidate Cur-loaded nanogels derived from salmon bone. We evaluate the potential contribution of Cur to nanogel structural organization. The molecular mechanisms of ionic cross-linking and multi-point hydrogen bonding were investigated *via* FT-IR and XRD, while morphology and size distribution were characterized using FE-SEM, HR-TEM, and DLS. Finally, the *in vitro* release kinetics were modelled, and the synergistic anti-inflammatory efficacy was validated in LPS-stimulated RAW 264.7 macrophage cell lines.

## 2 Materials and methods

### 2.1 Materials

The chondroitin sulfate (CS) was extracted and purified from the salmon bones as described in Section 2.2. Curcumin (≥95%) was purchased from Apollo Ingredients India Pvt. Ltd. Anhydrous calcium chloride (CaCl_2_, ≥96%), absolute ethanol, phosphate-buffered saline (PBS) components, and all necessary analytical-grade reagents were obtained from Merck Millipore. For the *in vitro* cell culture and biological evaluation, the RAW 264.7 murine macrophage cell line was obtained from the American Type Culture Collection (ATCC, Manassas, VA, USA). Dulbecco's Modified Eagle Medium (DMEM), fetal bovine serum (FBS), penicillin–streptomycin antibiotic cocktail, and the Griess reagent kit were purchased from Gibco, Thermo Fisher Scientific (Waltham, MA, USA). Lipopolysaccharide (LPS, from *Escherichia coli* O111:B4), dexamethasone (positive control, ≥98%), and 3-(4,5-dimethylthiazol-2-yl)-2,5-diphenyltetrazolium bromide (MTT) assay kit were purchased from Sigma-Aldrich Company.

### 2.2 Extraction of chondroitin sulfate

Fresh salmon bones were first boiled in distilled water (100 °C) for 30 minutes to remove adherent muscle tissue and residual lipids. The bones were then washed multiple times with warm water and dried at 60 °C until a constant weight was achieved. The dried bones were mechanically crushed into coarse fragments (approx. 1–5 mm) using a high-speed blender. To remove deeply embedded marrow fats that could interfere with the ionic cross-linking of the final nanogel, the powder was treated with an ethanol/acetone mixture (1 : 1 v/v) for 4 h, followed by final drying at 50 °C.

Chondroitin sulfate (CS) was isolated from obtained fish bone powder *via* enzymatic hydrolysis.^23,24^ Briefly, 22.32 g of dried bone powder was suspended in a bicarbonate-buffered solution (pH 8.0) and digested with papain (3.10 g) at 60 °C for 8 h. Following protein precipitation with TCA (pH 3.0) and subsequent centrifugation, the CS was recovered through cold ethanol fractionation (1 : 4 v/v). To purify the product, the crude CS was dissolved in 20 mL of distilled water. This solution was placed in dialysis tubing (MWCO 3.5 kDa) to remove low-molecular-weight impurities *via* dialysis against distilled water with periodic media changes. The dialyzed solution was concentrated to half volume and re-precipitated with ethanol (1 : 4 v/v). The final precipitate was centrifuged at 5000 rpm for 10 minutes and dried at 50 °C to obtain 0.94 g of purified CS. The extraction was done in triplicate and the yield of purified CS was 4.18 ± 0.25% calculated based on dried fish bone powder.

The chemical structure of the CS was confirmed by ^1^H-and ^13^C-NMR spectra using a Bruker AVANCE NEO 600 spectrometer (Bruker AXS, Germany) operating at 70 °C. Sample was prepared at a concentration of 20 mg mL^−1^ in D_2_O using 3-(trimethylsilyl)propionic-2,2,3,3-*d*_4_ acid sodium salt (DSS) as an internal reference. ^13^C-NMR spectrum was acquired with a spectral width of 38 000 Hz, a pulse width of 30°, a relaxation delay (*d*_1_) of 2.0 s, and an accumulation of 10 240 scans.

Uronic acid content of the CS was determined by following the carbazole method^25^ using d-glucuronic acid as a standard. Sulfate content was estimated using the gelatin/BaCl_2_ method^26^ after hydrolysis of CS in 2 M CF_3_COOH (TFA), 8 h at 100 °C. Protein and lipid contents were determined by the method of Bradford using bovine serum albumin as a ref. 27 and Bligh–Dyer method,^28^ respectively.

The weight-average molecular weight (*M*_w_) of the CS was determined *via* High-Performance Size-Exclusion Chromatography (HPSEC) on a Shimadzu LC-20 Series instrument (Shimadzu, Japan). Chromatographic elution was performed using 0.1 M LiNO_3_ as the mobile phase at a controlled temperature of 40 °C with a flow rate of 1.0 mL min^−1^ across PSS SUPREMA ultrahigh columns, calibrated against commercial Dextran standards ranging from 1 to 670 kDa.

### 2.3 Fabrication of CS-Cur nanogels

The fabrication of CS-Cur nanogels was achieved *via* a modified ionic gelation method.^29^ To determine the ranges for formulation parameters, specifically CaCl_2_ concentration, chondroitin sulfate (CS) concentration, and the Cur/CS ratio, a preliminary screening was initially conducted. Individual exploratory formulations were prepared in single runs to map the broad experimental domain and identify general performance trends regarding particle characteristics. Based on these exploratory trends, the most promising formulation condition (1.0 mg mL^−1^ CS, 0.5 mM CaCl_2_, and a 1 : 20 Cur/CS mass ratio) was subsequently selected and prepared in triplicates (*n* = 3) to ensure reliable data (Tables S1–S3, SI). Briefly, exactly 10 mL of aqueous CS solution (1.0 mg mL^−1^) was stirred at 600 rpm. Then, 0.5 mL of curcumin stock solution in absolute ethanol (1.0 mg mL^−1^) was added dropwise (yielding an ethanol concentration of 4.76% v/v). Ionotropic gelation was induced by the dropwise addition of 1.0 mL of 0.5 mM aqueous CaCl_2_ stock solution (resulting in a final CaCl_2_ concentration of approximately 0.044 mM) at a rate of 1 mL min^−1^ under continuous stirring for 3 h at 25 °C. The colloidal dispersion was centrifuged at 10 000 rpm for 20 min. The resulting pellet was washed twice with distilled water, redispersed in 5 mL of distilled water, and lyophilized at −58 °C under 0.37 mbar for 48 h (Thermo Scientific ModulyoD Freeze Dryer). The wash fractions and the supernatant were preserved for encapsulation efficiency analysis.

### 2.4 Encapsulation efficiency and drug loading capacity

The encapsulation efficiency (EE) and drug loading capacity (LC) of curcumin within the CS nanogels were determined *via* an indirect method by quantifying the amount of unencapsulated free drug.^30^ The wash fractions and the supernatant obtained in Section 2.3 was analyzed for curcumin content using an HPLC-DAD system (Agilent 1100) under the following conditions: chromatographic column: C-18 reversed-phase column. Mobile phase: water/acetonitrile (containing 0.1% formic acid), flow rate: 0.6 mL min^−1^, injection volume: 5 µL, detection wavelength *λ* = 425 nm; column temperature: 25 °C. A standard calibration curve of curcumin with regression coefficient *R*^2^ = 0.998 (Fig. S1, SI) was constructed using peak area integrations to calculate the concentration of free curcumin.

The total initial mass of curcumin introduced into the system and the total mass of the recovered dried nanogels were recorded. The encapsulation efficiency (EE, %) and loading capacity (LC, %) were calculated using eqn (1) and (2), respectively.1
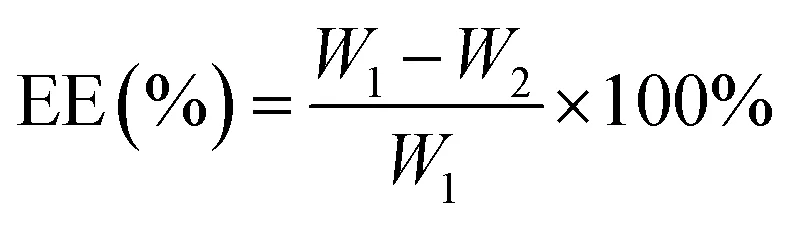
2
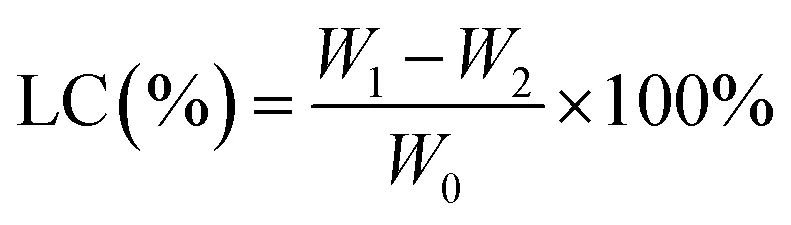
where: *W*_0_: total weight of dry nanogels recovered; *W*_1_: total amount of curcumin used and *W*_2_: free curcumin not encapsulated.

### 2.5 Physicochemical and morphological characterization

#### 2.5.1 FT-IR and XRD analysis

Fourier-transform infrared (FT-IR) spectra of native CS, free curcumin, blank nanogels, and CS-Cur nanogels were recorded on a Shimadzu FT-IR-8400S spectrometer (Shimadzu, Japan) within the wavenumber range of 4000–400 cm^−1^ utilizing the potassium bromide (KBr) pellet technique.

X-ray diffraction (XRD) profiles were acquired using a Bruker AXS D8 Advance diffractometer (Bruker, Germany) configured with Cu-Kα radiation (*λ* = 1.5406 Å) operating at 40 kV and 40 mA, scanned across a 2*θ* range of 5–30° with a step size of 0.02° to assess the amorphous-to-nanocrystalline phase transformations.

#### 2.5.2 DLS and electron microscopy (SEM/TEM) observations

The DLS and zeta-potential measurement were performed at 25 °C using a Litesizer 500 (Anton Paar, Austria) equipped with a 658 nm laser. Lyophilized nanogels were dispersed in distilled water at 500 ppm, with a measured pH of 7. Before analysis, samples were equilibrated at 25 °C for 60 s. Particle size and PDI were determined by DLS at 175°, with six automatically processed 10 s runs per measurement. Zeta potential was determined by ELS using the cmPALS technique. Each sample was independently measured in triplicate, the results are presented as mean ± SD (*n* = 3).

To evaluate colloidal behavior under physiological conditions, the lyophilized nanogels were redispersed in phosphate-buffered saline (PBS, pH 7.4) supplemented with 10% fetal bovine serum (FBS) as a physiologically relevant protein-containing medium at a concentration of 0.1 mg mL^−1^. The mixtures were incubated at 37 °C under gentle shaking. The particle size, PDI, and zeta potential of the nanogels were monitored over a period of 7 days to assess their resistance to serum protein-induced aggregation and structural integrity in biological fluids.

The surface topography of the nanogels was visualized using field-emission scanning electron microscopy (FE-SEM, JEOL JSM-IT800, Japan) operating at an accelerating voltage of 15 kV.

To estimate the actual core diameter and structural morphology of individual nanogels, Transmission Electron Microscopy (HR-TEM, JEM-2100, JEOL, Japan) was employed. A drop of the diluted nanogel solution was deposited onto a carbon-coated copper grid and allowed to air-dry completely at room temperature before observation at an accelerating voltage of 200 kV.

### 2.6 Swelling behavior determination

Swelling behavior of lyophilized blank CS nanogels and CS-Cur nanogels was investigated in a phosphate-buffered saline (PBS) solution at pH 7.4 to simulate physiological conditions. A known mass of pre-weighed lyophilized nanogels was submerged in the buffer solution and incubated at 37 °C for predetermined time intervals (4, 6, 8, 12, 24, and 48 h). At each time interval, the swollen nanogels were separated from the environmental buffer *via* centrifugation at 10 000 rpm for 10 min. The excess surface water on the tube walls was carefully removed by blotting with filter paper, and the weight of swollen nanogels was immediately recorded.

The equilibrium swelling index (SI) of the nanogels was quantified using the following mathematical relationship (eqn (3)).^26^3
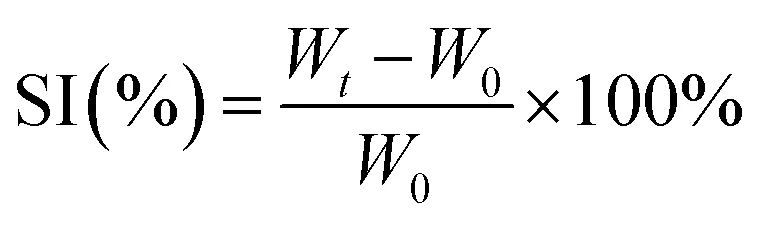
where: *W*_*t*_ represents the weight of the nanogels at a specific swelling time *t*, and *W*_0_ denotes the initial weight of the dry lyophilized nanogels.

### 2.7 *In vitro* release and kinetics modeling

The *in vitro* release behavior of curcumin-loaded nanogels was evaluated using the dialysis membrane method.^31,32^ Specifically, exactly 5.0 mg of lyophilized nanogels was redispersed in 1.0 mL of distilled water and transferred into a dialysis tubing bag (MWCO 3.5 kDa). The bag was immersed in 100 mL of phosphate-buffered saline (PBS, pH 7.4) containing 0.5% (w/v) sodium dodecyl sulfate (SDS) to ensure sink conditions by enhancing the solubility of curcumin. The release system was maintained at 37 °C with horizontal shaking at 100 rpm. At predetermined time intervals (1, 2, 4, 8, 12, 24, 36, 48, and 72 h), 1 mL of the release medium was withdrawn and replaced with 1 mL of fresh buffer. The cumulative mass of the released curcumin was quantified using the HPLC-UV method described in Section 2.4. A control group consisting of an equivalent amount of free curcumin in a dialysis bag was tested in parallel. Additionally, the chemical stability of curcumin in the release medium was verified by incubating free curcumin at 37 °C for 72 h, showing less than 5% degradation, confirming the reliability of the release data.

To mathematically elucidate the underlying mass transport mechanisms, the experimental drug release data (up to 60% of cumulative release) were fitted into four conventional mathematical models: zero-order, first-order, Higuchi and Korsmeyer–Peppas models. The equations for these models are expressed as follows:

4 • Zero-order model: *M*_*t*_ = *M*_0_ + *k*_0_*t*

5 • First-order model: ln(*M*_∞_ − *M*_*t*_) = ln *M*_∞_ − *k*_1_*t*

6 • Higuchi model: *M*_*t*_ = *k*_H_*t*^0.5^

7
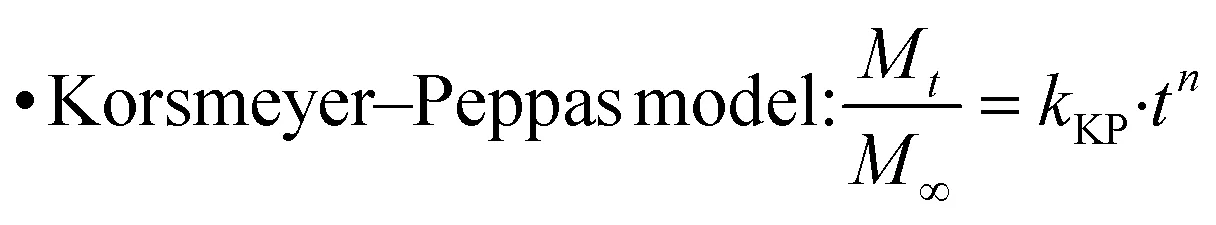
where *M*_*t*_/*M*_∞_ represents the fractional release of the drug at time *t*; *k*_0_, *k*_1_, *k*_H_, and *k*_KP_ are the respective release rate constants; and *n* is the diffusional exponent indicative of the specific transport mechanism. For pseudo-spherical nanogels, *n* = 0.43 mathematically corresponds to Fickian diffusion, while 0.43 < *n* < 0.85 indicates anomalous (non-Fickian) transport driven by coupled processes of both diffusion and polymer matrix relaxation.^33^

### 2.8 *In vitro* anti-inflammatory activity and cytotoxicity assays

#### 2.8.1 Quantification of nitric oxide (NO) production

The anti-inflammatory performance of the engineered carriers was evaluated by quantifying the inhibition of nitric oxide (NO) production in lipopolysaccharide (LPS)-stimulated RAW 264.7 murine macrophage cell lines.^34,35^ The cells were seeded in 96-well plates at a density of 2 × 10^5^ cells per well and cultured in Dulbecco's Modified Eagle Medium (DMEM) supplemented with 10% fetal bovine serum (FBS) and 1% penicillin–streptomycin at 37 °C under a humidified atmosphere containing 5% CO_2_.

To induce an inflammatory response, the cells were stimulated with 1 µg mL^−1^ of LPS in the presence or absence of varying concentrations of free curcumin, blank CS, and CS-Cur nanogel for 24 h. Note that the mass of the added nanogels was calibrated based on the predetermined loading capacity (LC) value to ensure the absolute curcumin concentration strictly corresponded to that of the free curcumin groups. A physical mixture of blank CS and free curcumin approximately 24 : 1 (CS : Cur, w/w), matching the 4% loading capacity, was also evaluated. Dexamethasone was used as a positive control. The accumulation of nitrite, a stable oxidative end-product of NO, in the culture supernatant was determined using the colorimetric Griess reagent kit. Briefly, equal volumes (100 µL) of cell culture supernatant and Griess reagent were mixed in a fresh 96-well plate and incubated at room temperature for 10 min in the dark. The absorbance was measured at 540 nm using a microplate reader (BioTek Elx800).

The IC_50_ values were calculated using non-linear regression analysis *via* TableCurve 2Dv4 software. The percentage inhibition of NO production was quantified using the following formula (eqn (8)):8
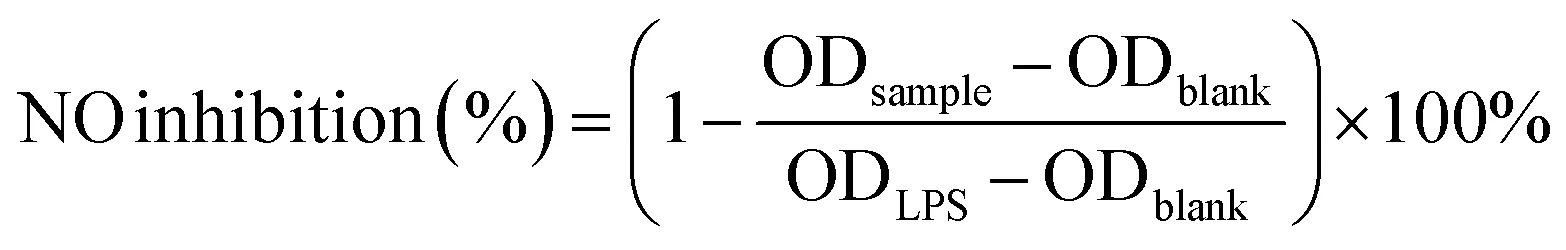


#### 2.8.2 Cytotoxicity assessment *via* MTT assay

The viability of RAW 264.7 cells after treatment was evaluated using the 3-(4,5-dimethylthiazol-2-yl)-2,5-diphenyltetrazolium bromide (MTT) assay to confirm that the observed anti-inflammatory activity was not an artifact of cellular toxicity.^36,37^

Following the collection of the culture supernatant for NO quantification, the remaining adherent cells in each well were replenished with 90 µL of fresh culture medium and 10 µL of MTT solution (5 mg mL^−1^). After a 4 hours incubation period at 37 °C, the medium was carefully discarded, and the formed purple formazan crystals inside the viable cells were dissolved by adding 100 µL of 100% DMSO to each well.

The optical density (OD) was subsequently measured at a wavelength of 540 nm using a BioTek Elx800 microplate reader. The percentage of cell viability was calculated relative to the untreated control groups using the following formula (eqn (9)):9
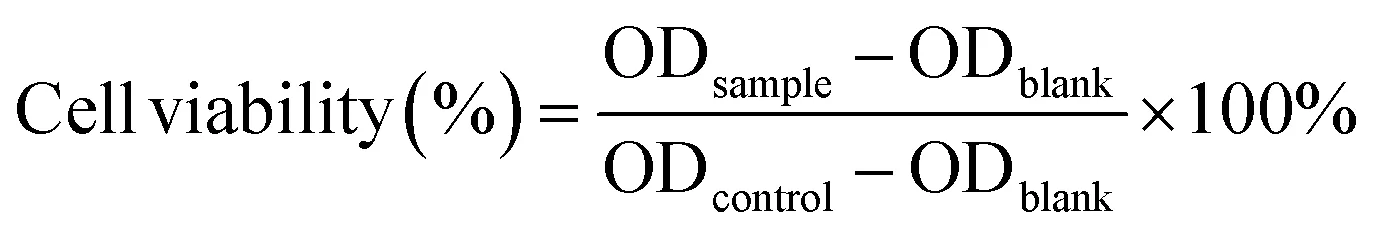
where: OD_sample_, OD_blank_, and OD_control_ represent the absorbance of the treated samples, the reagent blank (well without cells), and the untreated control group (cells without LPS and active components), respectively.

#### 2.8.3 Statistical analysis

All experiments were performed as three independent experiments, each with three technical replicate wells (*n* = 3 independent experiments), and the data are expressed as mean ± standard deviation (SD). A two-way analysis of variance (ANOVA) followed by Dunnett's *post hoc* test was applied to determine statistical differences among groups, where the free curcumin was designed as the control group. Statistical significance was defined as *p < 0.05, **p < 0.01, ***p < 0.001 and ****p < 0.0001.

## 3 Results and discussion

### 3.1 Structural characteristics of extracted chondroitin sulfate

The structural characteristics of the extracted chondroitin sulfate (CS) were elucidated using ^1^H-NMR and ^13^C-NMR spectroscopies. The spectra (Fig. 1) exhibited typical spectral characteristics of marine-derived sulfated glycosaminoglycans, structurally composed of alternating β-d-glucuronic acid (GlcA) and *N*-acetyl-d-galactosamine (GalNAc) disaccharide repeating units.

**Fig. 1:**
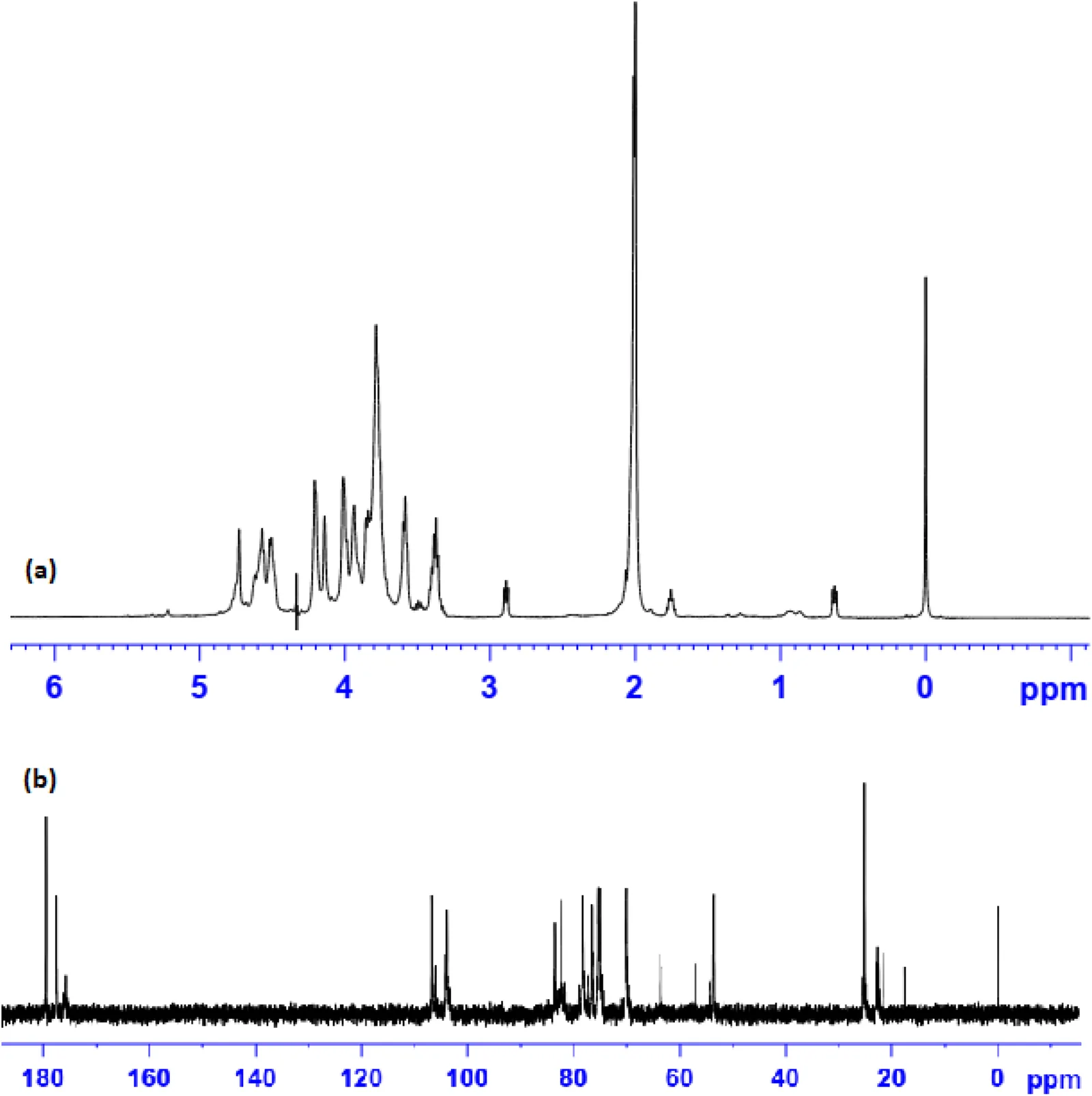
(a) ^1^H-NMR spectrum and (b) ^13^C-NMR spectrum of chondroitin sulfate extracted from salmon bone.

The ^1^H-NMR spectrum of CS (Fig. 1a) displays well-resolved signals corresponding to the carbohydrate backbone. Signals observed between 4.50 ppm and 4.57 ppm are assigned to the anomeric protons (H-1) of the β-d-GlcA and β-d-GalNAc residues. The minor splitting and multiplicity in this region reflect the structural heterogeneity regarding the sulfation patterns along the polymer chain. The ring proton region (3.35–4.21 ppm), which contains a prominent sharp signal at 4.01 ppm along with multiplets (3.93–4.14 ppm), is highly characteristic of the H-4 proton adjacent to the *O*-sulfate group in GalNAc units, suggesting a significant presence of 4-*O*-sulfation (chondroitin 4-sulfate, CS-A type). The remaining overlapping multiplets from 3.40 ppm to 3.90 ppm correspond to the H-2, H-3, and H-5 protons of the pyranose rings. A highly intense singlet at 2.01 ppm is attributed to the methyl protons (–CH_3_) of the *N*-acetyl group (–NHCOCH_3_) in the GalNAc units.

The ^13^C-NMR spectrum of CS (Fig. 1b) provides higher structural resolution, confirming the functional groups of the CS structure. The resonance at 175.71 ppm is assigned to the carbonyl carbon of the *N*-acetyl group (–NHCOCH_3_). The split signals at 177.44 ppm and 179.49 ppm correspond to the carboxylate carbons (–COO^−^) of the GlcA units. This splitting denotes distinct local electrostatic environments, likely induced by variations in neighboring sulfation positions (C-4 and C-6). In anomeric region (101–107 ppm), the signals at 106.68 ppm, 106.12 ppm, and 104.05/103.90 ppm are attributed to the anomeric carbons (C-1) involved in the β-1,3 and β-1,4 glycosidic linkages. The coexistence of multiple anomeric peaks strongly corroborates that the salmon bone CS is a hybrid structure consisting of both chondroitin 4-sulfate (CS-A) and chondroitin 6-sulfate (CS-C) isomers. Signals between 75 ppm and 84 ppm are associated with ether-linked or deshielded carbons due to sulfation, where the peaks at 77.35–78.27 ppm are characteristic of the sulfated C-4 of GalNAc. A peak at 53.60 ppm is assigned to the C-2 of GalNAc, which shifts upfield due to the directly attached nitrogen atom (–CH–NHAc). The sharp signal at 25.18 ppm represents the methyl carbon (–CH_3_) of the *N*-acetyl group, in good agreement with the ^1^H-NMR data.^38,39^ The sulfation patterns were confirmed by cross-peaks H4/C4 of CS-A and H6/C6 of CS-C in HSQC spectra (Fig. S2, SI). The detailed signal assignments were provided in Table S4 (SI).

In conclusion, the NMR spectra confirmed the structural identity of the extracted CS, revealing a co-existence of CS-A and CS-C variants. Notably, this specific distribution of CS-A and CS-C isomers serves as a distinct structural signature for the salmon bone-derived CS examined in this study. Furthermore, this hybrid CS-A/CS-C composition provides an abundance of sulfated and carboxylated sites that actively facilitate cooperative ionic interactions during nanogel formation.

Complementing the structural elucidation by NMR, the quantitative purity of the extracted CS were systematically evaluated. The results indicated that residual protein and lipid contents in the purified CS were 0.10 ± 0.02% (w/w) and 0.31 ± 0.02% (w/w), respectively, verifying the high purity of the extracted material. Furthermore, total sulfate and uronic acid contents of the CS were determined as 20.6 ± 0.8% and 33.2 ± 1.1%, respectively, aligning well with the characteristic composition of marine-derived chondroitin sulfate.^23^

The weight-average molecular weight (*M*_w_) was determined by HPSEC to be 36.9 kDa. This specific molecular weight provides a favorable chain length regime, which is reasonably suggested to support effective macromolecular packing and stable junction zone formation during ionic gelation.

### 3.2 Chemical coordination and crystalline state of CS-Cur nanogels

FTIR spectroscopy was employed to evaluate the structural integrity of the individual components, investigate the ionic interactions and assess incorporation of curcumin (Cur) to the chondroitin sulfate (CS) nanogel matrix.

The spectrum of native CS (Fig. 2a) exhibited a broad absorption band at 3342 cm^−1^, corresponding to the stretching vibrations of hydroxyl (OH) and amine (NH^−^) groups. The peaks at 1602 cm^−1^ and 1412 cm^−1^ were assigned to the asymmetric and symmetric stretching vibrations of carboxylate (–COO^−^) groups, respectively. Additionally, the sharp band at 1226 cm^−1^ represented the S=O stretching of the sulfate groups (–SO_3_^−^), while the peak at 854 cm^−1^ confirmed the C–O–S axial sulfation of CS.^40,41^

**Fig. 2:**
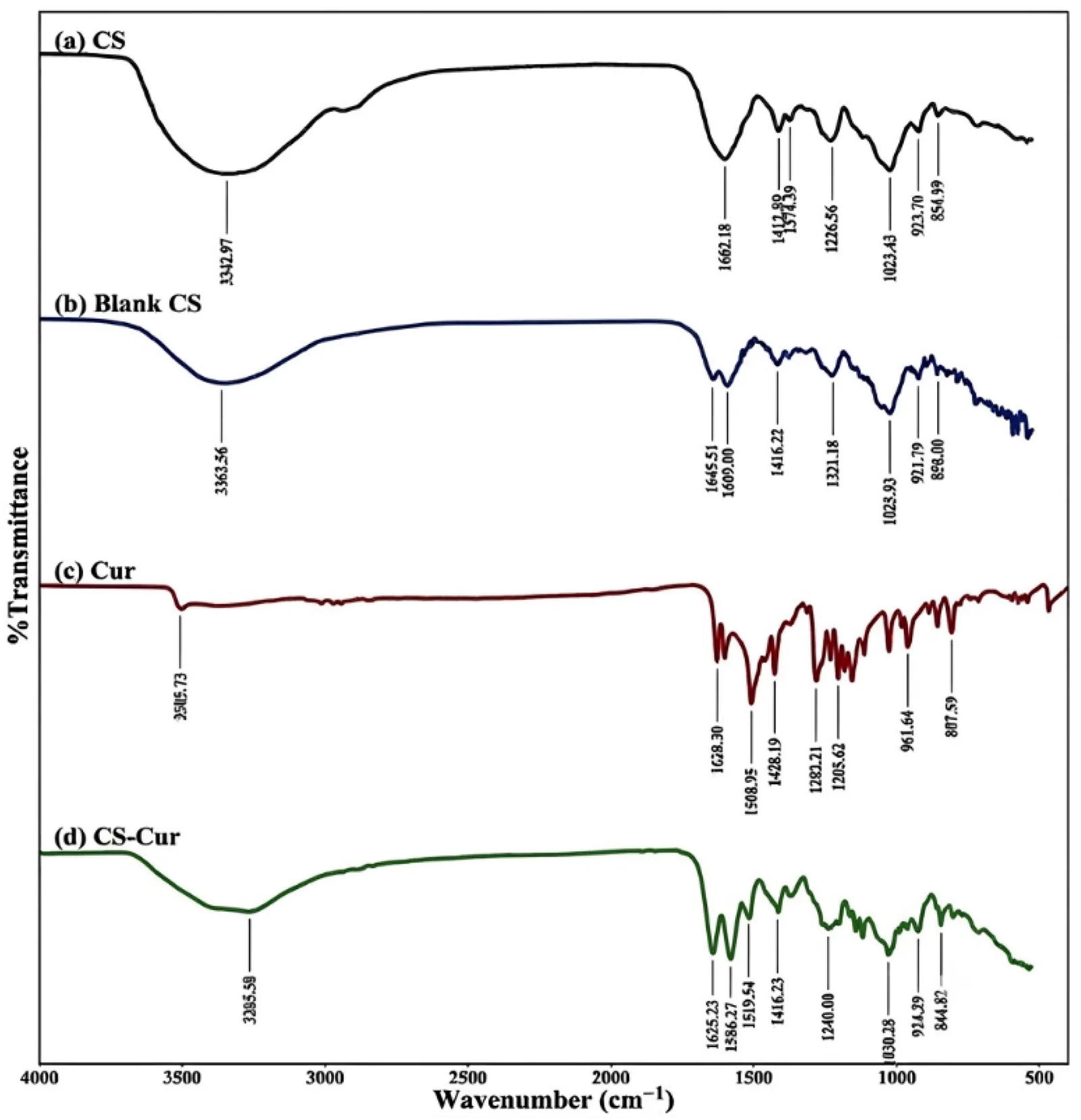
FTIR spectra (a) CS; (b) blank CS; (c) curcumin; and (d) CS-Cur nanogels.

Upon ionotropic gelation to form the blank CS nanogels, the spectrum (Fig. 2b) underwent highly specific modifications that reveal the cross-linking mechanism. The broad OH and NH-stretching band shifted to 3362 cm^−1^, indicating the change in the hydrogen bonding networks upon cage formation. Concurrently, the primary carboxylate peak at 1602 cm^−1^ shifted to 1609 cm^−1^ and developed a shoulder at 1645 cm^−1^. This shift, combined with the band-splitting effect, indicates that the native local symmetry of the COO^−^ groups was significantly perturbed by the coordination with Ca^2+^ ions. Furthermore, the symmetric stretching vibration of the carboxylate groups (COO^−^) shifts from 1412 cm^−1^ to 1416 cm^−1^. These spectral shifts suggest the involvement of COO^−^ groups in coordinating with Ca^2+^ ions, supporting the formation of a crosslinked ionic network within the nanogel framework.^42,43^ In contrast, the sulfate band displayed only a minor shift from 1226 cm^−1^ to 1223 cm^−1^ along with a slight broadening. This suggests that while the core cross-linked network is driven predominantly by carboxylate chelation, the sulfate groups are suggested to be preferentially oriented toward the aqueous phase, potentially providing an electrostatic shielding layer that imparts colloidal stability to the nanogels.^43–45^

For pure Cur, a highly sharp, crystalline spectral fingerprint was observed (Fig. 2c). Key diagnostic bands included the sharp phenolic –OH stretch at 3505 cm^−1^, the conjugated aromatic C=O and C=C stretching at 1628 cm^−1^, and the skeletal benzene ring breathing vibration at 1508 cm^−1^.^40^ Following the incorporation of Cur into the nanogel matrix, the resulting CS-Cur nanogel spectrum (Fig. 2d) demonstrated a successful dual-driven encapsulation mechanism involving both hydrophobic driving forces and multi-point hydrogen bonding. First, the –OH absorption band shifted from 3362 cm^−1^ (in the blank nanogel) to 3265 cm^−1^. This redshift is consistent with the phenolic OH groups of Cur, once localized inside the carrier, participating in enhanced intermolecular hydrogen bonding with the hydrophilic hydroxyl and residual amine groups of the CS matrix. Second, the reappearance of aromatic peak at 1519 cm^−1^ together with the pronounced broadening and red shift of the hydroxyl stretching band supports the successful incorporation of curcumin into the CS nanogel network.

In summary, the FTIR data indicated a sequential assembly process: a stable nanogel matrix is first driven by localized ionotropic carboxylate cross-linking, which subsequently provides a protective, hydrogen-bonded hydrophobic pocket that successfully molecularly entraps the curcumin molecules.

To further determine whether the encapsulated curcumin remained crystalline or underwent a solid-state transition after encapsulation, XRD analysis was performed. The XRD patterns of native CS, pure Cur, blank CS nanogels, and Cur-loaded CS nanogels (CS-Cur) are shown in Fig. 3.

**Fig. 3:**
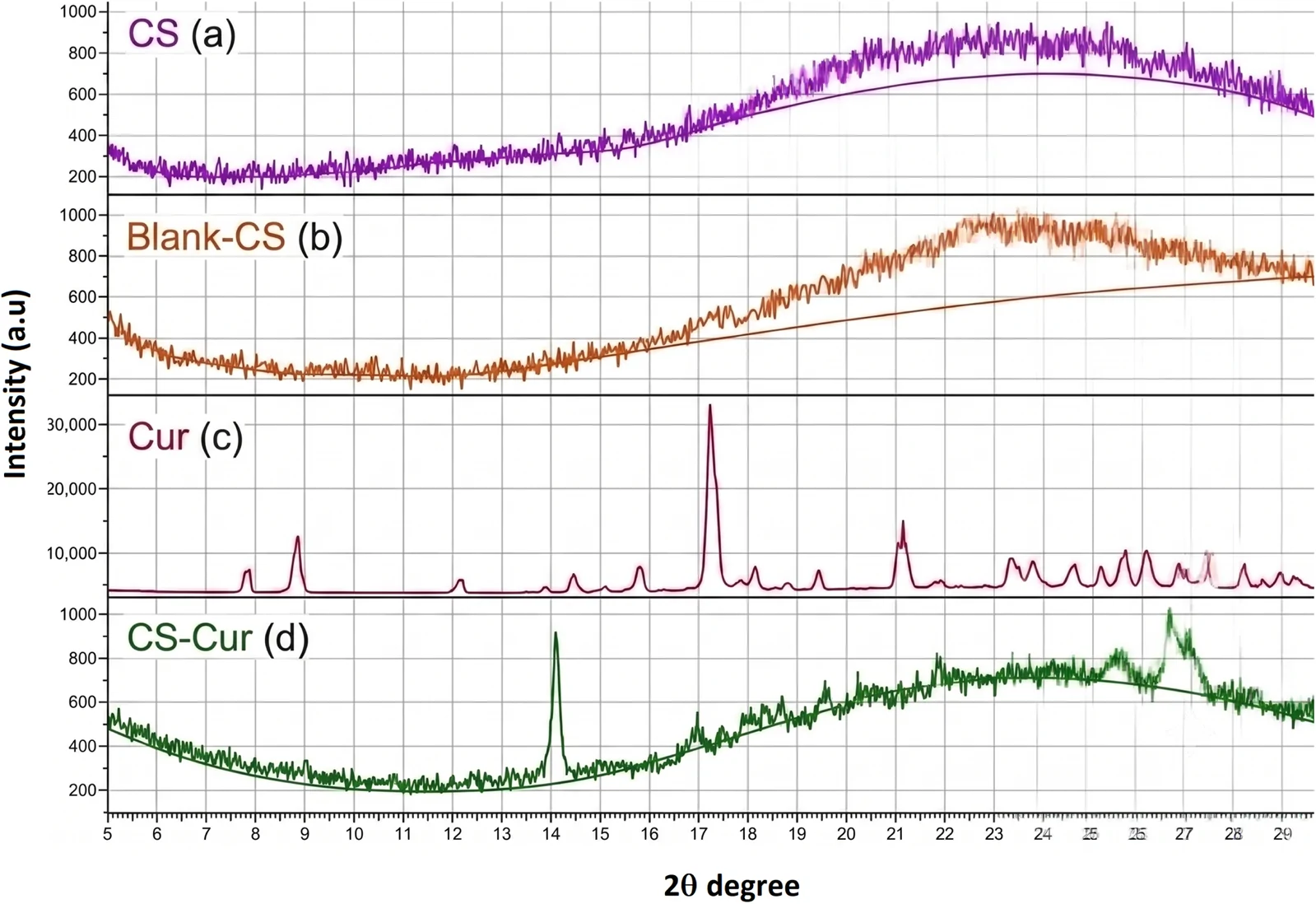
XRD pattern of CS (a); blank CS (b); curcumin (c); and CS-Cur nanogels (d). Note that the intensity axis for curcumin (c) is scaled to 30 000 counts to display its crystalline peaks, while other samples (a, b and d) are presented at a scale of 1000 counts to highlight their amorphous nature.

The XRD pattern of native CS (Fig. 3a) displays a broad, poorly defined diffraction reflection centered at a 2*θ* value of approximately 22–24°. This feature is indicative of the predominantly disordered, amorphous macromolecular nature of the native CS chains.^46^

Upon ionic cross-linking to form the blank CS nanogels (Fig. 3b), the diffractogram retains a typical amorphous state. The preservation of this amorphous halo demonstrates that the ionic gelation process restricts polymer chain mobility into a rigidized, disordered three-dimensional network rather than inducing crystallization.

In contrast, the diffraction pattern of pure Cur (Fig. 3c) exhibits intense, sharp reflections. These sharp peaks represent a highly ordered, crystalline state characteristic of native polymorphic curcumin crystals.^47,48^

For the CS-Cur nanogels (Fig. 3d), a dramatic transformation is observed in the XRD profile. The crystalline reflections characteristic of pure Cur have completely vanished, being replaced by a dominant, broad amorphous background scattering similar to the CS matrix. This complete disappearance of the drug's crystalline peaks provides strong evidence of successful encapsulation. During the ionic-induced assembly of the nanogels, the lipophilic curcumin molecules are molecularly entrapped within the interior hydrophobic nanovoids of the cross-linked CS matrix. This spatial restriction prevents the Cur molecules from self-associating or stacking into an ordered lattice, thereby forcing the drug to transition into a highly dispersed, amorphous molecular state.

Interestingly, the XRD pattern of the CS-Cur nanogels displays a newly emerged, sharp reflection at 2*θ* approx. 14°, which was entirely absent in the amorphous profile of the blank CS nanogel. The appearance of this peak suggests a localized structural reorganization of curcumin within the nanogel matrix, indicating that the encapsulated components have adopted a more ordered arrangement, potentially stabilized by specific interactions with the CS polymer chains. This phenomenon may be attributed to restricted crystallization dynamics occurring within the matrix environment. While the formation of specific curcumin polymorphic states, comparable to those reported by Sanphui *et al.*^47^ (where reflections around 2*θ* ≈ 14° were identified), serves as a plausible contributing factor, alternative mechanisms such as polymer-induced structural ordering or confinement-related spatial constraints cannot be ruled out. While differentiating these concurrent factors requires further in-depth investigation, these observations collectively highlight a distinct local structural organization induced within the nanogel system.

By correlating the FT-IR and XRD findings, a mechanism of structural stabilization and drug-matrix compatibility can be deduced. The significant broadening and red-shift of the –OH stretching vibration to 3265 cm^−1^ in the FT-IR spectrum, coupled with the emergence of the distinct reflection at 2*θ* approx. 14° in the XRD profile, collectively indicate that curcumin does not merely act as a passive therapeutic cargo. Instead, it actively engages in extensive intermolecular interactions with the CS matrix. This interplay between electrostatic ionic crosslinking and enhanced intermolecular hydrogen-bonding/hydrophobic interactions restricts the molecular mobility and ensures high entrapment stability. In summary, these findings strongly support that curcumin is effectively hosted within the stable, ionically cross-linked chondroitin sulfate nanogel matrix. While the majority of the encapsulated curcumin exists in an amorphous molecular dispersion, the XRD reflection at 14° indicates that a minor fraction of the encapsulated material retains or develops a locally ordered state within the nanogel microenvironment.

### 3.3 Size, surface charge, and morphological characterization of CS-Cur nanogels

The size distribution, surface charge, and morphology of the curcumin-loaded chondroitin sulfate nanogels were evaluated using DLS, zeta potential analysis, FE-SEM, and HR-TEM.

As shown in Fig. 4a, the CS-Cur nanogels exhibited a mean hydrodynamic diameter (*D*_h_) of 217.3 ± 15.6 nm with a polydispersity index (PDI) of 0.25 ± 0.02. This PDI value indicates a relatively narrow and acceptable particle size distribution for an ionically cross-linked polysaccharide nanogel system. The obtained hydrodynamic size also confirms that the Ca^2+^-mediated ionic gelation process was able to generate nanoscale colloidal particles without extensive aggregation. The surface charge of the nanogels was further examined by zeta potential analysis. As presented in Fig. 4b, the CS-Cur nanogels showed a negative zeta potential of −26.9 ± 0.14 mV. This negative surface charge can be attributed to the remaining non-cross-linked carboxylate and sulfate groups of the CS chains on the nanogel surface. The magnitude of the zeta potential suggests good electrostatic stabilization, which may help reduce particle aggregation in aqueous dispersion.

**Fig. 4:**
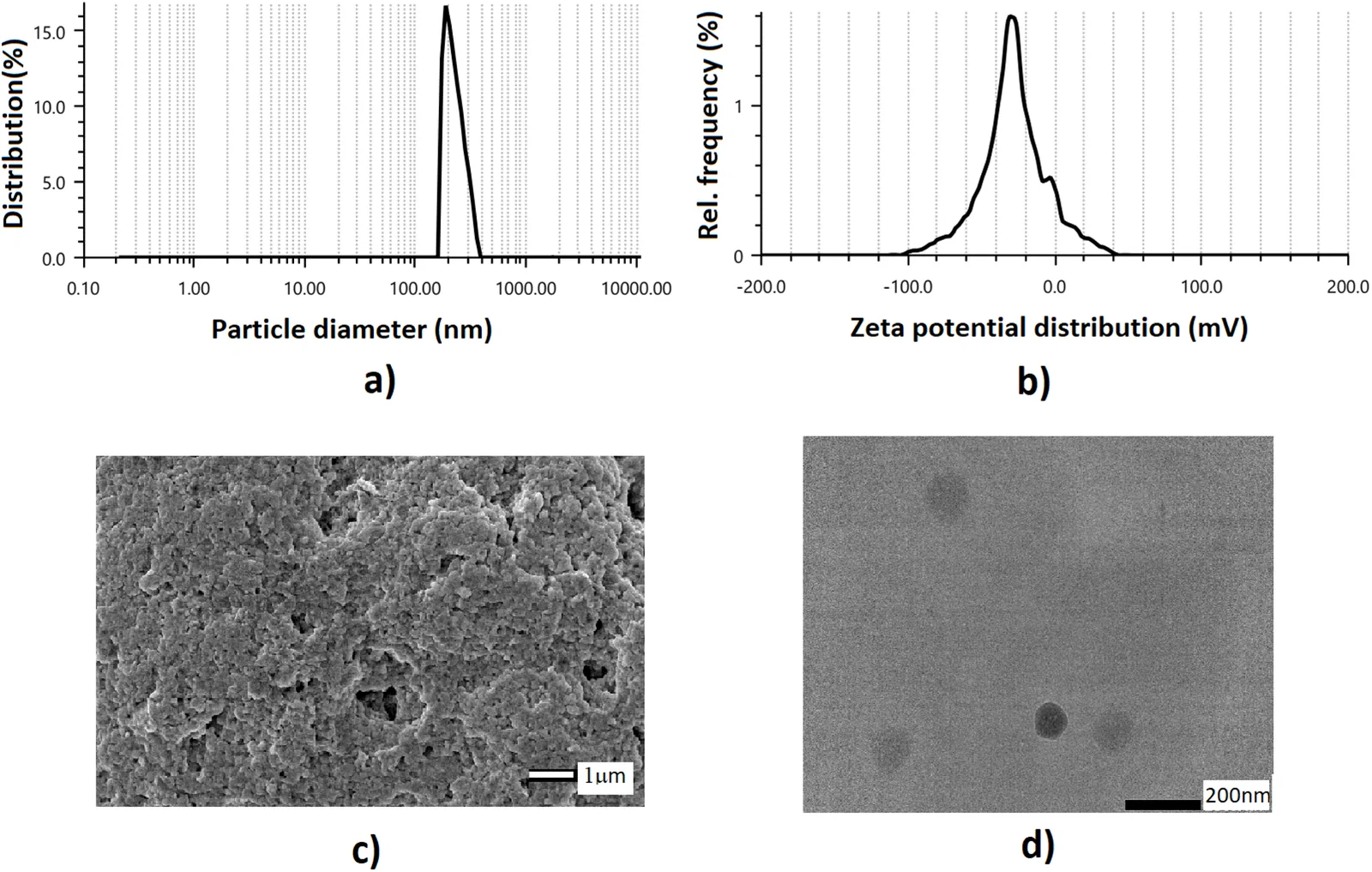
Physicochemical and morphological characterization of the CS-Cur nanogels: (a) particle size distribution determined by DLS; (b) zeta potential distribution curve; (c) FE-SEM image showing the interconnected porous network of the lyophilized state, and (d) HR-TEM micrograph highlighting the distinct spherical structures in the dispersed state.

The colloidal stability of the nanogels in biological environments is a critical prerequisite for potential biomedical applications. To investigate their behavior under physiologically relevant conditions, the nanogels were incubated in PBS (pH 7.4) supplemented with 10% FBS as a physiologically relevant protein-containing medium at 37 °C. As measured by DLS over a 7 days period, the nanogels maintained a stable hydrodynamic diameter (221.5 ± 12.4 nm), a narrow PDI (0.27 ± 0.02) and a persistent negative zeta potential (−25.4 ± 0.8 mV) in the PBS-FBS medium. These results demonstrate good colloidal stability and high resistance to serum protein-induced aggregation, indicating that the electrostatic repulsion provided by the charged groups on the CS matrix likely contributes to resistance against particle agglomeration in complex biological fluids.

The morphological transformation during the fabrication of CS-Cur nanogels was evaluated by FE-SEM. The native CS displays a typical bulk, irregular, and layered sheet-like morphology (Fig. S3a, SI), while pure Cur exhibits distinct, highly defined, and large rod-like crystalline blocks (Fig. S3b, SI).^48^ Upon the ionic gelation process, a morphological transformation occurs; the CS-Cur nanogels displayed a porous and interconnected network structure rather than completely isolated spherical particles (Fig. 4c). This morphology is characteristic of hydrophilic polysaccharide-based hydrogels after lyophilization, where water removal and capillary forces induce partial collapse and association of the cross-linked polymer chains.^49,50^ The disappearance of the initial layered sheets of chondroitin sulfate and prismatic crystals of curcumin in the final formulation provides compelling evidence of the successful self-assembly and the molecular dispersion of curcumin within the cross-linked chondroitin sulfate matrix.

Finally, HR-TEM was employed to elucidate the internal structure and dispersity of the nanogels. TEM observation deposited from diluted dispersion revealed discrete, nearly spherical nanoparticles with relatively uniform morphology (Fig. 4d). The dry particle diameter estimated from the TEM micrographs was approximately 100–150 nm, which was smaller than the hydrodynamic diameter determined by DLS. This difference is expected for hydrogel-based nanoparticles because DLS measures the fully hydrated particles together with their solvated polymer chains and hydration shell, whereas TEM records the dehydrated and collapsed structure under high-vacuum conditions. The core–shell-like contrast observed in several particles may reflect the formation of denser inner domains within the cross-linked CS matrix. Together with the FT-IR and XRD results, these morphological observations support the successful incorporation of curcumin into the ionically cross-linked CS nanogel network. Overall, the combined DLS, zeta potential, SEM, and TEM results demonstrate that the CS-Cur nanogels form stable nanoscale hydrogel particles with a hydrated size suitable for colloidal drug delivery applications.

### 3.4 Encapsulation efficiency and *in vitro* drug release of CS-Cur nanogels

The fabricated CS-Cur nanogels exhibited an encapsulation efficiency (EE) of 73.55 ± 2.45%, which underscores the robust entrapment capability of the ionic gelation network. The rapid coordination between Ca^2+^ ions and the anionic groups (–COO^−^ and –OSO_3_^−^) of CS effectively formed a Ca^2+^-mediated crosslinked network that encapsulated Cur before macro-phase separation could occur. Concurrently, the loading capacity (LC) was found to be 4.06 ± 0.31%. This controlled loading may prevent the aggregation or crystallization of hydrophobic drug molecules within the highly hydrophilic polysaccharide network, thereby preserving a molecularly dispersed state.

Swelling ability is a fundamental property for hydrogel-based drug delivery systems, as it directly dictates solvent penetration, matrix relaxation, and the subsequent diffusion-controlled release of encapsulated therapeutics. The swelling behavior (SI %) of the CS-Cur nanogels was monitored over 48 hours at physiological pH (7.4) (Fig. 5a). As shown in the swelling profile, the nanogels exhibited a rapid water uptake during the initial 4 hours (SI approximately 50.5%), followed by a gradual increase and reaching an equilibrium plateau at approximately 12 to 48 hours, with a maximum swelling index of 85.1 ± 6.2%. Compared to the blank CS nanogels (which reached a higher equilibrium swelling index of 112.0 ± 5.5%), the CS-Cur nanogels showed a noticeably restricted swelling capacity. This restricted swelling is attributed to the encapsulation of hydrophobic curcumin and its secondary non-covalent interactions (such as hydrogen bonding and hydrophobic forces) within the polymer matrix, which further compacts the Ca^2+^- crosslinked ionic network and reinforces its structural stability. Consequently, this supports the hypothesis that curcumin contributes to network compaction through secondary interactions. Beyond the 12 hours threshold, the swelling rate plateaued, yielding equilibrium SI values of approximately 84% at 24 h and 85% at 48 hours. This stabilization behavior marks the attainment of a thermodynamic equilibrium between the osmotic swelling pressure (driven by the electrostatic repulsion of unreacted –SO_3_^−^ and –COO^−^ groups) and the elastic contractive forces of the ionically cross-linked network.

**Fig. 5:**
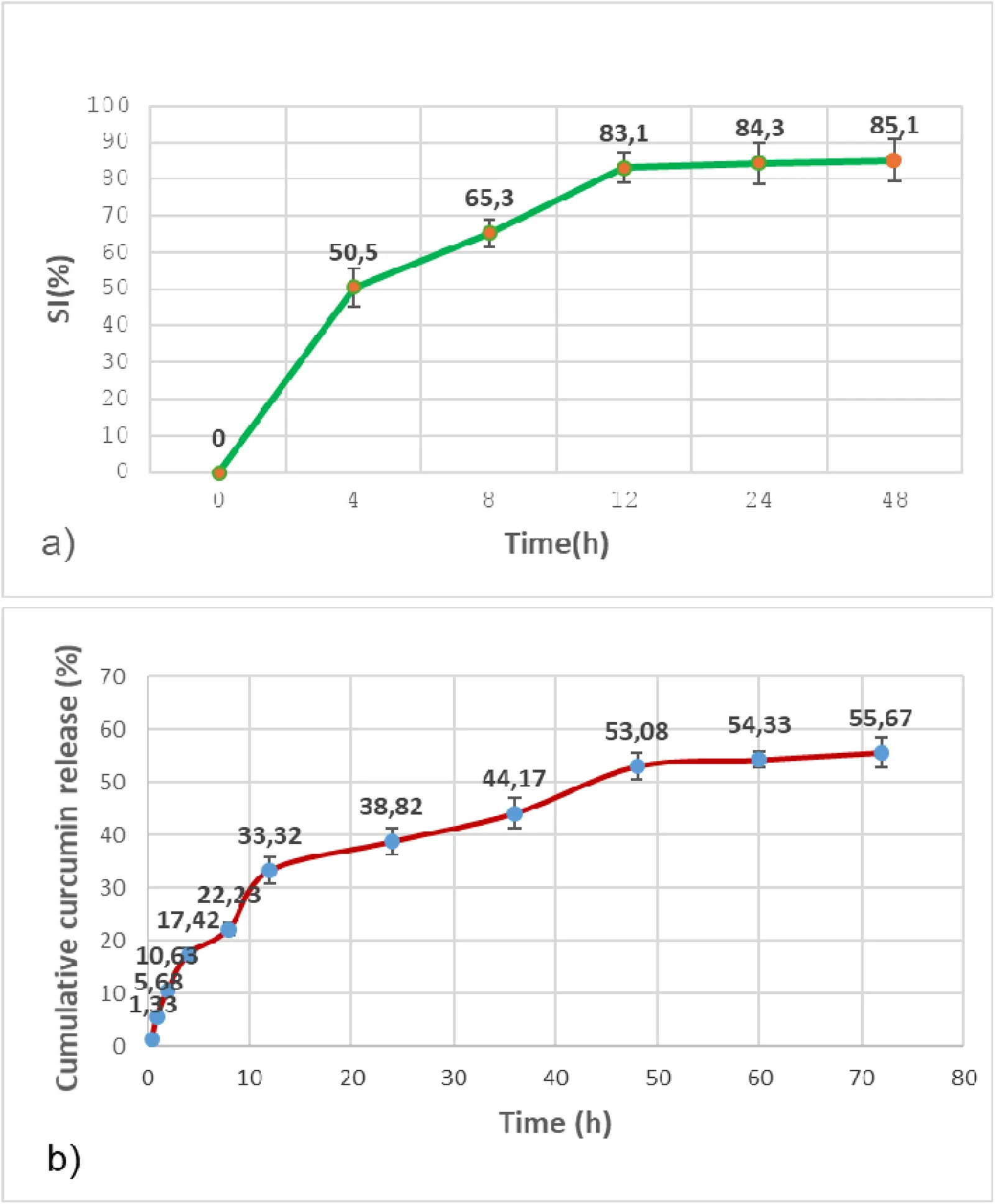
Swelling index (a) and *in vitro* curcumin release profile (b) of CS-Cur nanogels in PBS (pH 7.4) at 37 °C.

To assess the controlled-release performance of the CS-Cur nanogels, *in vitro* studies were conducted under sink conditions in PBS (pH 7.4) supplemented with 0.5% SDS to account for the limited aqueous solubility of curcumin. The control group (free curcumin) exhibited a rapid dialysis recovery, exceeding 85% within the first 8 hours, which confirms that the dialysis membrane itself does not impose a significant barrier to drug diffusion. In contrast, the CS-Cur nanogels displayed a sustained biphasic release profile, as discussed below.

The system exhibited a biphasic release profile, characterized by an initial controlled burst followed by a prolonged, sustained release phase (Fig. 5b). In the first phase (0–12 h), a relatively rapid dissolution profile was observed, with the cumulative curcumin release reaching approx. 33.32% at the 12 hours mark. This initial burst effect is primarily governed by two parallel phenomena: (i) the rapid dissolution and diffusion of curcumin molecules that are weakly adsorbed or entrapped near the surface layer of the CS matrix. (ii) The immediate penetration of the buffer medium into the hydrophilic outer shell, inducing rapid swelling of the non-cross-linked segments of the polysaccharide. Subsequently, the second phase (12–72 h) transitions into a highly controlled, sustained release rate, with the cumulative percentage slowly ascending to 55.67% at 72 hours. The mass transport mechanism in this sustained phase is strictly regulated by the interconnected hydrogel network ionically secured by Ca^2+^ bridges.

The remaining approx. 44% of the curcumin is primarily attributed to strong hydrophobic interactions and physical entrapment within the dense Ca^2+^-crosslinked chondroitin sulfate matrix, which restricts immediate diffusion. This retained fraction is tightly embedded within less accessible domains and may require longer release times and/or progressive matrix relaxation for further release.

Consequently, the system functions as an effective controlled-release carrier, where the remaining payload may require longer release times and/or progressive matrix relaxation. Such sustained release behavior may be advantageous for reducing rapid exposure to high drug concentrations and prolonging drug availability.

To elucidate the release mechanism of curcumin from the CS-Cur nanogels, the cumulative release data were analyzed using four widely recognized kinetic models: zero-order, first-order, Higuchi, and Korsmeyer–Peppas.

The linearized equations and the corresponding correlation coefficients (*R*^2^) for each model are summarized in Table S5. Among the tested models, the Korsmeyer–Peppas model exhibited the highest correlation coefficient (*R*^2^ = 0.995), followed by the Higuchi (*R*^2^ = 0.988), first-order (*R*^2^ = 0.985), and zero-order (*R*^2^ = 0.973) models. Specifically, the calculated release constant (*k*) was 0.051, while the diffusion exponent (*n*) was found to be 0.711. For spherical polymeric matrices, an *n* value within the range of 0.43 < *n* < 0.85 is indicative of an anomalous transport mechanism (non-Fickian diffusion).^33^ The superior fit of this model confirms that the drug release is not governed by a single mechanism; instead, the release behavior is characterized by drug diffusion coupled with the swelling and relaxation of the chondroitin sulfate-based matrix. This demonstrates that the marine-derived CS framework provides a dynamic barrier that effectively regulates the sustained release of curcumin over the observed 72 hours period.

Mechanistically, this behavior is dictated by the spatial architecture of the CS matrix. Upon immersion in the buffer, the peripheral, less-crosslinked hydrophilic domains of the nanogel undergo rapid hydration and expansion, driving the initial phase of curcumin release. However, as the hydration front advances inward toward the core, the transport process becomes restricted by the dense, localized Ca^2+^-coordinated junction zones established *via* ionic bridges. The entrapped curcumin molecules, which exist in a highly dispersed amorphous state as indicated by XRD, must simultaneously undergo molecular diffusion through these dense polymer networks and winding nano-channels while the polymer matrix undergoes slow structural relaxation. This dual-controlled mechanism effectively prevents any catastrophic initial burst release, ensuring that the therapeutic drug is metered out in a steady, highly controlled, and biphasic pattern over 72 hours period.

### 3.5 Biological efficacy and anti-inflammatory activity

To evaluate the biomedical potential and therapeutic safety of the fabricated nano-delivery system, the biocompatibility and *in vitro* anti-inflammatory activity of native CS, free Cur, the physical mixture of blank CS + free Cur, and CS-Cur nanogels were investigated. The anti-inflammatory efficacy was validated by evaluating their capacity to inhibit nitric oxide (NO) production in LPS-stimulated RAW 264.7 macrophages in a dose-dependent manner. The cytotoxicity was evaluated using the MTT assay. The results were shown in Table 1 and Fig. 6.

**Table 1:** Anti-inflammatory of CS-based systems in LPS-stimulated RAW 264.7 cells

Sample	NO inhibition (%) at a screening dose of 20 µg mL^−1^^a^	IC_50_ for NO^a^ (µg mL^−1^)
Blank CS	10.36 ± 1.1	85.59 ± 4.52
Free curcumin	42.55 ± 1.37	21.05 ± 2.33
Mix of CS + curcumin	33.47 ± 1.29	46.24 ± 5.04
CS-Cur nanogel	59.78 ± 1.64	15.86 ± 0.8
Dexamethasone (positive control)	54.15 ± 1.35 (at 20 µM)	12.86 ± 1.41 (µM)

a Data represent mean ± SD (*n* = 3, independent replicates).

**Fig. 6:**
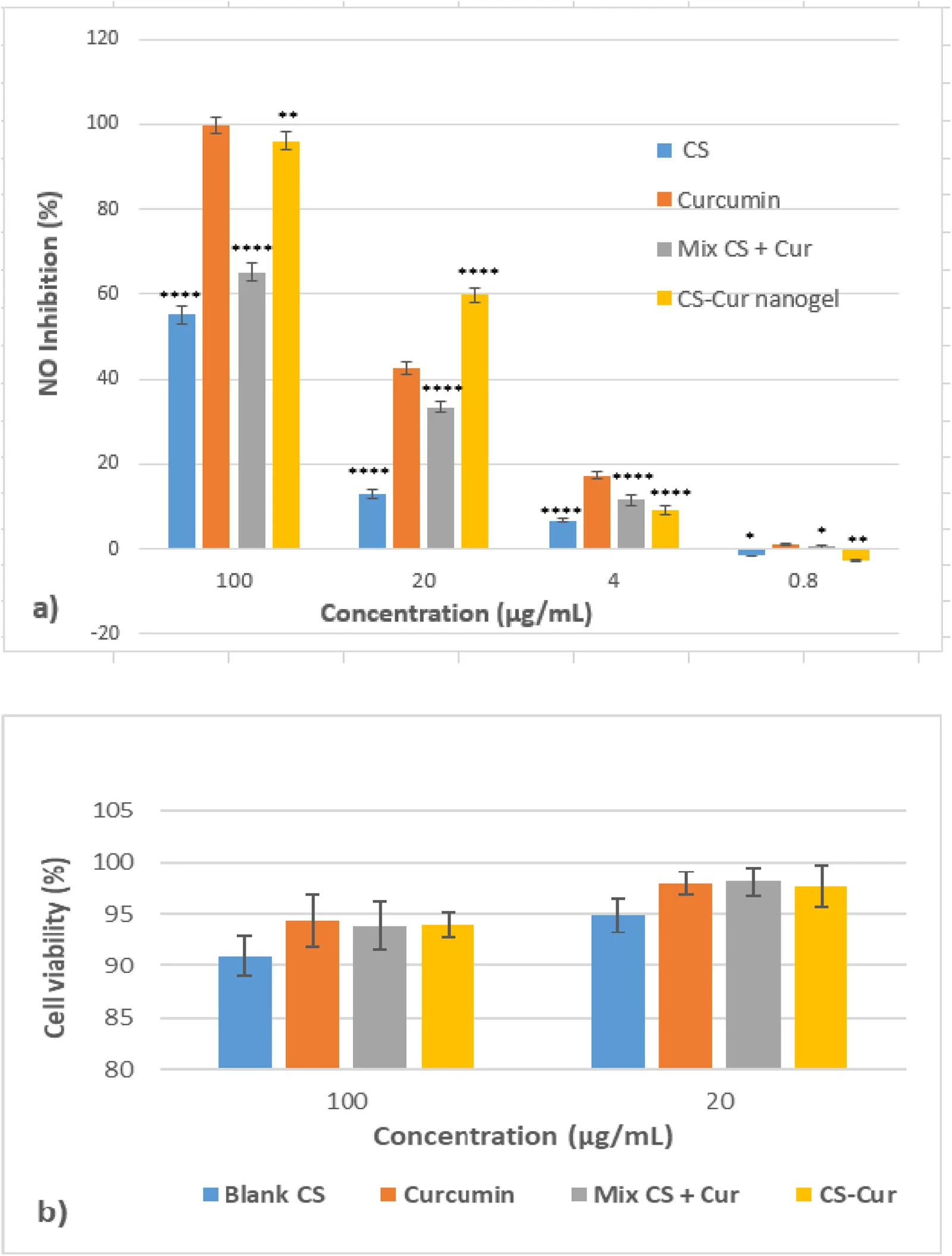
(a) Inhibitory effects on nitric oxide (NO) production and (b) cell viability of RAW 264.7 macrophages treated with blank CS, free curcumin, physical mixture of CS + curcumin (Mix CS + Cur), and CS-Cur nanogel. Data represent mean ± SD (*n* = 3, independent replicates). Statistical significance in (a) relative to the curcumin control group is denoted as: *p < 0.05, **p < 0.01, and ****p < 0.0001.

As summarized in Table 1, the blank CS exhibited relatively weak intrinsic anti-inflammatory activity, with IC_50_ of 85.59 ± 4.52 µg mL^−1^. This high IC_50_ value logically correlates with its minimal NO inhibition percentage (10.36 ± 1.1%) observed at the sub-threshold screening concentration of 20 µg mL^−1^. To illustrate this dose-dependent behavior, the concentration-dependent profiles (ranging from 0.8 to 100 µg mL^−1^) were monitored and illustrated in Fig. 6a. As expected, blank CS initiated dose-dependent suppression across the tested range, reflecting its intrinsic bioactivity.

This therapeutic effect is highly characteristic of marine-derived polysaccharides, where the specific sulfation patterns and high density of negatively charged functional groups (sulfate and carboxyl groups) are postulated to modulate cell surface receptors or impede pro-inflammatory cytokine cascades. This baseline biological activity highlights a distinct marine advantage, consistent with previous findings by Waiprib *et al.*,^39^ who leveraged CS extracted from tuna heads to fabricate nanocarriers with enhanced bio-functionality.

Upon curcumin encapsulation, the CS-Cur nanogels demonstrated an enhancement in anti-inflammatory performance, yielding a lower IC_50_ value of 15.86 ± 0.8 µg mL^−1^ compared to that of free curcumin (21.05 ± 2.33 µg mL^−1^) and the physical mixture (46.24 ± 5.04 µg mL^−1^). At a treatment dose of 20 µg mL^−1^, the nanogels suppressed 59.78 ± 1.64% of NO generation (*p* < 0.0001). Meanwhile, the physical mixture of CS and Cur exhibited an intermediate efficacy (*p* < 0.0001 at 20 µg mL^−1^). This supports the interpretation that the enhanced activity is associated with nanoformulation rather than simple physical combination of the two components. Regarding the positive control, dexamethasone exhibited strong anti-inflammatory activity, as evidenced by an NO inhibition percentage of 54.15 ± 1.35% at a screening concentration of 20 µM and a calculated IC_50_ value of 12.86 ± 1.41 µM. These significant values align with previous literature and formally validate the responsiveness and reliability of the implemented *in vitro* inflammatory model.^51,52^

This pronounced therapeutic amplification can be attributed to the structural and physicochemical characteristics of the nanogel matrix. Firstly, free curcumin is hydrophobic and prone to rapid hydrolytic degradation at physiological pH, restricting its effective presence. The encapsulation within the marine CS framework improves the aqueous solubility and dispersion stability of curcumin, thereby facilitating its effective availability in the culture medium. This enhancement aligns perfectly with recent findings where marine-derived CS nanoparticles significantly improved the sustained release and solubility of lipophilic cargos.^32^ Within the Ca^2+^ -crosslinked CS framework, curcumin is entrapped in a molecularly dispersed amorphous state (as corroborated by the XRD results), which accelerates its solubility in the culture medium. Secondly, the hydrophilic polysaccharide shell acts as a protective shield against premature chemical degradation, ensuring a sustained and controlled release of the cargo. Once released, the dynamic structure of the CS matrix, governed by a non-Fickian transport mechanism, ensures the sustained availability of curcumin in the medium. Therefore, the observed superior bioactivity stems from a combined effect of enhanced payload stability and the intrinsic anti-inflammatory properties inherent to the salmon bone-derived chondroitin sulfate matrix. Importantly, the MTT cell viability assay conducted concurrently on the remaining adherent cells confirmed that the cell viability remained above 90% across all treated concentrations (Fig. 6b). This critically rules out the possibility that the reduction of NO levels was caused by generalized cellular toxicity or cell death, indicating that the reduced NO production was not attributable to treatment-induced cytotoxicity.

## 4 Conclusions

A Ca^2+^- crosslinked nanogel platform based on salmon bone-derived chondroitin sulfate was successfully developed for the controlled delivery of curcumin. Structural characterization demonstrated that ionic coordination and hydrogen bonding govern nanogel assembly, while curcumin encapsulation substantially alters its solid-state organization within the polymer network. These findings support the concept that curcumin may contribute to nanogel structural organization rather than acting solely as a therapeutic payload. These structural changes are closely associated with sustained drug release and enhanced anti-inflammatory activity without detectable cytotoxicity. Beyond demonstrating the feasibility of a sustainable marine-derived carrier, this study highlights the importance of cargo-induced structural organization in regulating the performance of polysaccharide nanogels, providing a useful framework for the rational design of future hydrophobic drug delivery systems. Building upon these foundational materials-science insights, broader molecular mechanism evaluations and quantitative cellular internalization assays are currently underway in our ongoing follow-up studies.

## Author contributions

Conceptualization: T. T. T. T.; formal analysis: T. T. T. T., Q. V. N., N. C. N., H. T. P., C. D. H., T. T. M. Q.; investigation: T. T. T. T.; resources: Q. V. N., writing – original draft: T. T. T. T., N. C. N.; H. T. P.; writing – review & editing: T. T. T. T., Q. V. N.; funding acquisition: T. T. T. T.; project administration: T. T. T. T.; all authors have read and agreed to the current version of the manuscript.

## Conflicts of interest

There are no conflicts to declare.

## Supplementary Material

RA-016-D6RA06378K-s001

## Data Availability

The datasets generated and analyzed during this study are available from the corresponding author upon reasonable request. Supplementary information (SI): preliminary formulation screening data, NMR assignments for chondroitin sulfate, drug release kinetic modelling and FE-SEM images of chondroitin sulfate and curcumin. See DOI: https://doi.org/10.1039/d6ra06378k.
