# Supplementary material for: Salmon bone-derived chondroitin sulfate nanogels for controlled delivery of curcumin: release behavior, structural characteristics and anti-inflammatory efficacy
Source: RSC Adv. 2026 Oct 5;16(46):60118–29. doi: 10.1039/d6ra06378k (PMC13636292; doi:10.1039/d6ra06378k)
Supplement: Supplementary file 1 [file RA-016-D6RA06378K-s001.pdf]

Supplementary Information (SI) for RSC Advances.
This journal is © The Royal Society of Chemistry

Supplementary Information

Salmon Bone-Derived Chondroitin Sulfate Nanogels for Controlled Delivery of Curcumin: Release Behavior, Structural Characteristics and Anti-Inflammatory Efficacy

Quang Van Ngo,[a] Nguyen Chi Nguyen,[b] Hong Thi Pham,[b] Cuong Duc Ho,[b] Thu Thi Minh Quach,[a] and Thuy Thi Thu Thanh*[a]

a. Institute of Chemistry, Vietnam Academy of Science and Technology, 18 Hoang Quoc Viet, Nghia Do, Hanoi 100000, Vietnam.

b. School of Chemistry and Life Science, Hanoi University of Science and Technology. 01 Dai Co Viet, Bach Mai, Hanoi, 100000, Vietnam

** Corresponding author: E-mail: thuyttt@ich.vast.vn.; ORCID: 0000-0003-0535-947X*

Table S1. Influence of Crosslinker $CaCl_2$ Concentration

Cur/CS	Cur con. (mg/ml)	CS conc. (mg/ml)	$CaCl_2$ conc. (mM)	Particle Size (nm)	Zeta Potential (mV)	PDI
1:20	1	1	1	228	-27.0	0.48
1:20	1	1	0.5	217.3 ± 15.6	-26.9 ± 0.14	0.25 ± 0.02
1:20	1	1	0.2	378	-25.5	0.27

As tabulated in Table S1, a crosslinker ($CaCl_2$) concentration of 0.5 mM was locked as the most favourable condition, yielding the minimum hydrodynamic size of 217.3 ± 15.6 nm and a highly narrow size distribution (PDI = 0.25 ± 0.02). Deviations to higher or lower ionic concentrations directly induced poor ionotropic gelation control or undesirable macro-particle aggregation.

Table S2. Influence of CS Concentration

Cur/CS	Cur con. (mg/ml)	CS conc. (mg/ml)	$CaCl_2$ conc. (mM)	Particle Size (nm)	Zeta Potential (mV)	PDI
1:20	1	1	0.5	217.3 ± 15.6	-26.9 ± 0.14	0.25 ± 0.02
1:20	1	0.5	0.5	676	-21.4	0.25
1:20	1	2	0.5	1001	-25.1	0.20

Variations in CS concentration significantly altered the self-assembled nanogel matrix density (Table S2). A concentration of 1.0 mg/mL provided the ideal polymer network density required for the formation of stable nano-vehicles with appropriate colloidal features (size of 217.3 ± 15.6 nm).

Table S3. Influence of Cur/CS mass Ratio

Cur/CS	Cur con. (mg/ml)	CS conc. (mg/ml)	$CaCl_2$ conc. (mM)	Particle Size (nm)	Zeta Potential (mV)	PDI
1:10	1	1	0.5	656	-19.1	0.47
1:20	1	1	0.5	217.3 ± 15.6	-26.9 ± 0.14	0.25 ± 0.02
1:40	1	1	0.5	495	-26.3	0.59

The Cur/CS mass ratio of 1:20 demonstrated the most favorable colloidal parameters, the selected condition for the balance between hydrophobic cargo loading and a high negative zeta potential -26.9 ± 0.14 mV), which guarantees long-term electrostatic stability.

Note. Data for the screening formulations represent single run (n = 1) to identify system trend. Data for the selected formulation represent the mean ±SD of three independent runs (n = 3).

Table S4. ^{1}H and ^{13}C NMR chemical shift assignments for chondroitin sulfate extracted from salmon bone

Residue	C/H Position	^{13}C Chemical Shift (ppm)	^{1}H Chemical Shift (ppm)
GlcA			
	C1 / H1	104.05 / 103.90	4.50 – 4.52
	C2 / H2	73.33 – 74.78	3.38 – 3.42
	C3 / H3	75.11 – 75.58	3.57 – 3.59
	C4 / H4	78.27 – 82.48	3.71 – 3.78
	C5 / H5	76.58	3.69
	C6	177.44 / 179.49	—
GalNAc			
	C1 / H1	106.12 / 106.68	4.55 – 4.73
	C2 / H2	53.60	3.93 – 3.98
	C3 / H3	77.35 – 82.48	3.82 – 3.85
	C4 / H4	77.35 (for CS-A)<br>69.91 (for CS-C)	4.70 – 4.78<br>4.10 – 4.15
	C5 / H5	70.18 – 71.35	3.69 – 3.71
	C6 / H6	63.68 (for CS-A)<br>68.50 (for CS-C)	3.74 – 3.81<br>4.10 – 4.21
	-C=O	175.71	—
	$-CH_3$	25.18	2.01

Table S5. Linearized equations and correlation coefficients R^2 of four models

	Zero-order	First-order	Higuchi	Korsmeyer-Peppas
Eq.	$Mt = 2.563t + 2.126$	$\ln(100 - Mt) = -0.034t + 4.586$	$Mt = 10.211t^{0.5} – 6.367$	$\ln(Mt/M_\infty) = 0.711\log(t) – 1.291$
R^2	0.973	0.985	0.988	0.995

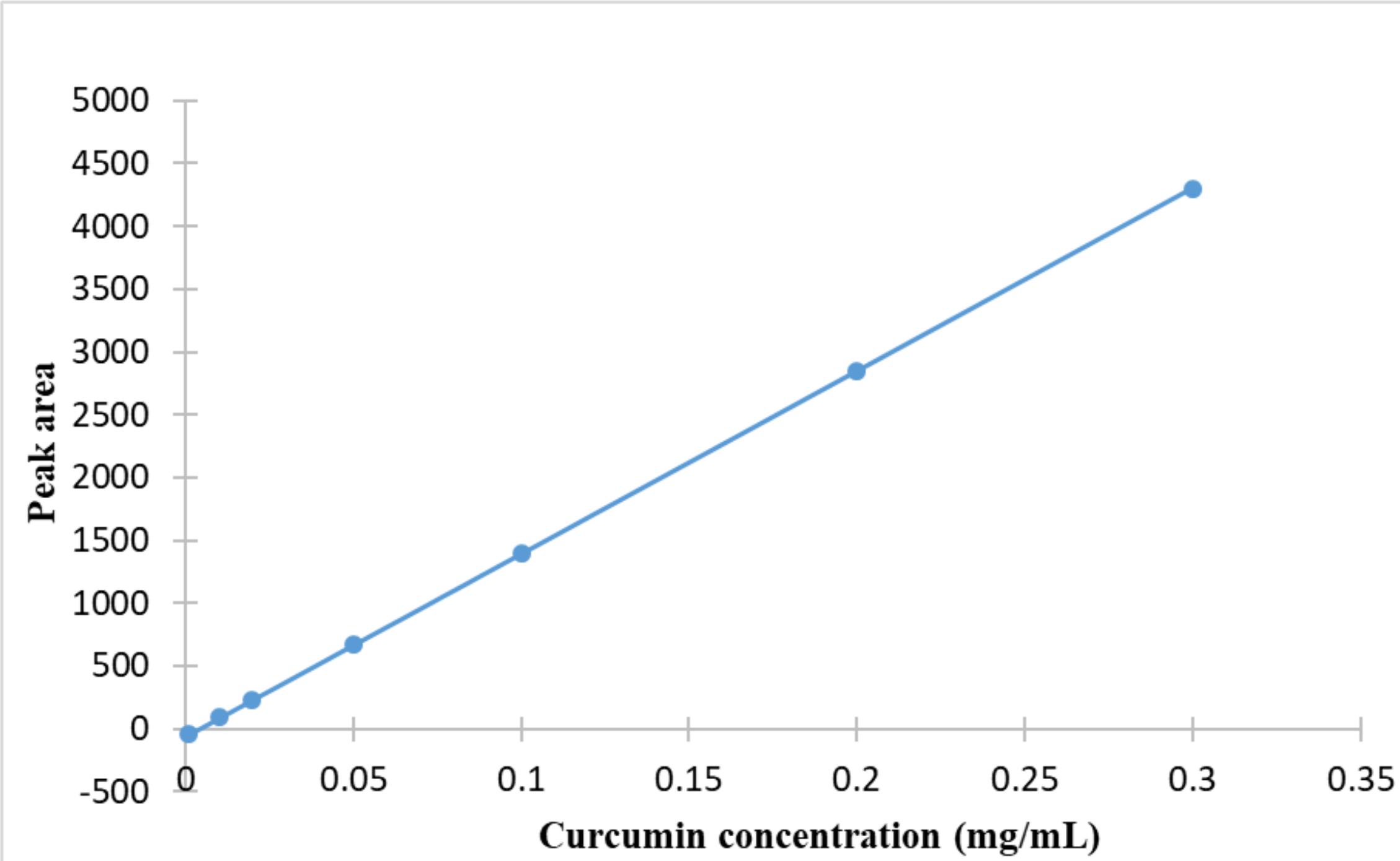

Fig. S1 Standard calibration curve of curcumin exhibited excellent linearity, typically expressed as y = 14525x – 57 (x = concentration; y = peak area), with a correlation coefficient R^2 of 0.998.

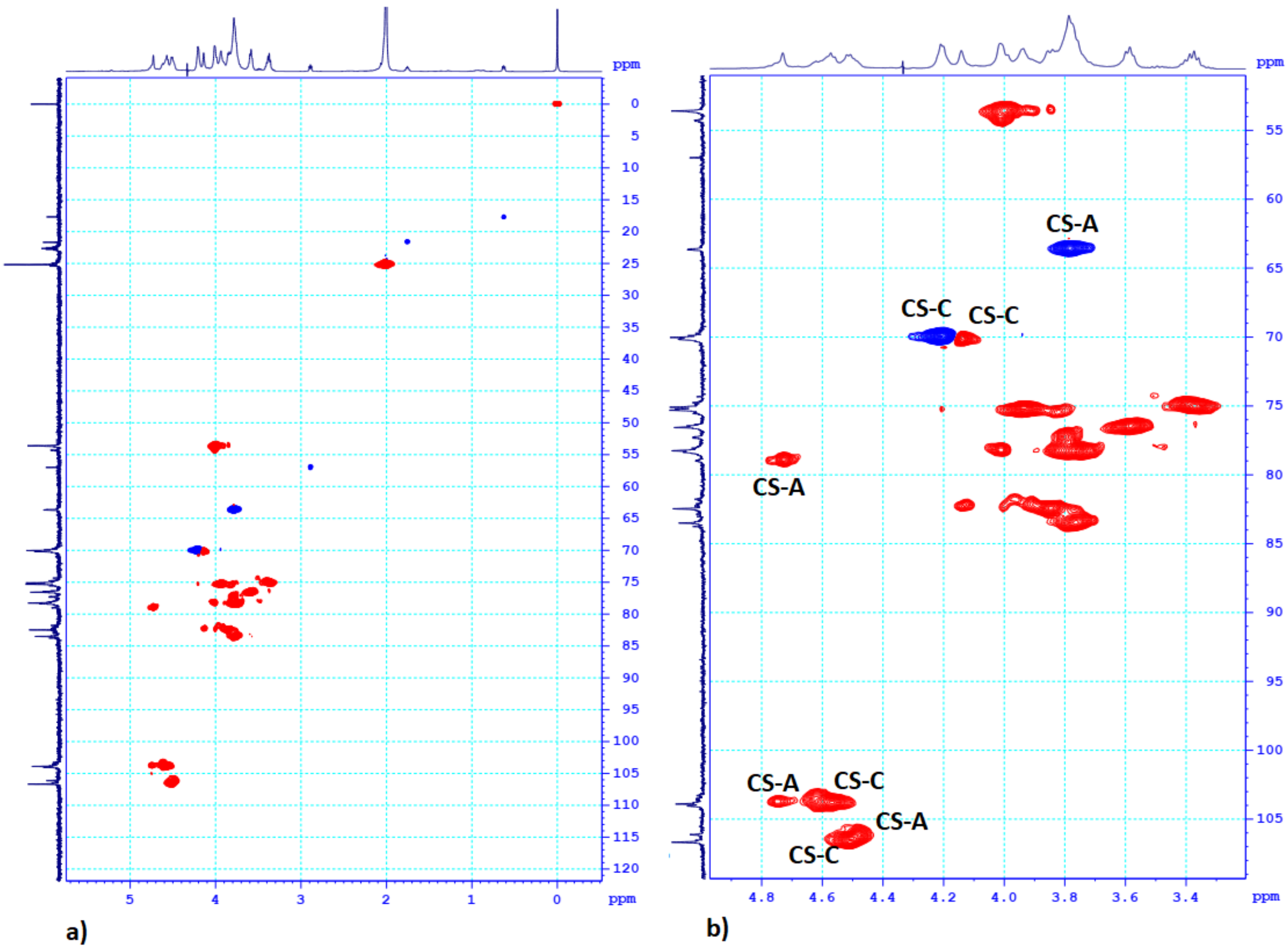

Fig. S2 HSQC a) Full spectrum b) Expanded spectrum of chondroitin sulfate extracted from salmon bone. The cross-peaks confirm CS-A and CS-C variants

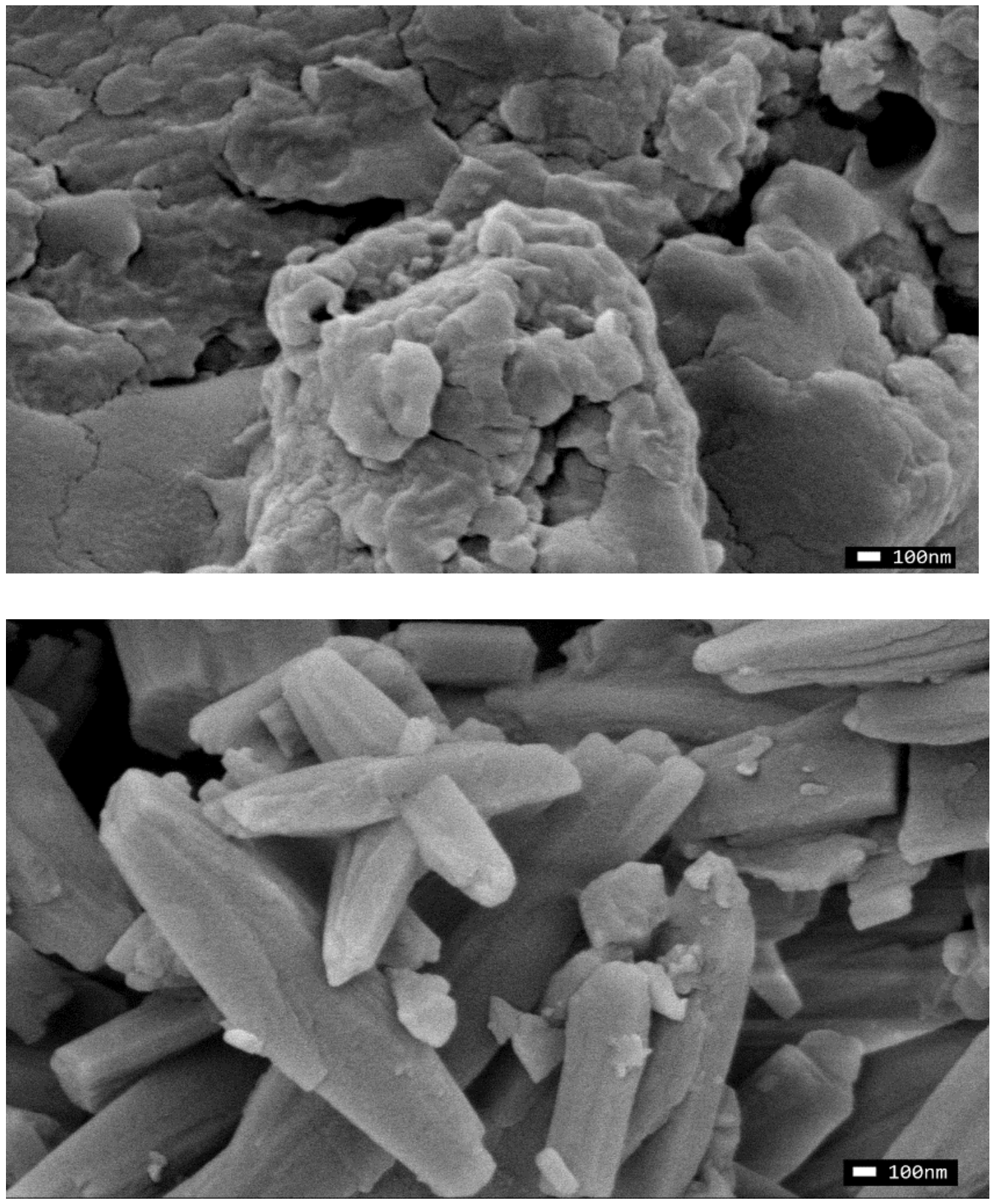

a)

b)

Fig. S3 (a) FE-SEM image of native chondroitin sulfate showing the bulk, irregular, and layered sheet-like morphology, and (b) FE-SEM image of pure curcumin highlighting the rod-like crystalline blocks
